# Understanding Socio-cultural Influences on Smoking among Older Greek-Australian Smokers Aged 50 and over: Facilitators or Barriers? A Qualitative Study

**DOI:** 10.3390/ijerph120302718

**Published:** 2015-03-02

**Authors:** Masoud Mohammadnezhad, George Tsourtos, Carlene Wilson, Julie Ratcliffe, Paul Ward

**Affiliations:** 1 Discipline of Public Health, Flinders University, GPO Box 2100, Adelaide, South Australia 5001, Australia; E-Mails: george.tsourtos@flinders.edu.au (G.T.); carlene.wilson@flinders.edu.au (C.T.); paul.ward@flinders.edu.au (P.W.); 2 Department of Health Education and Promotion, School of Public Health, Tehran University of Medical Sciences, Tehran 1414743777, Iran; 3 Centre for Clinical Change and Health Care Research, Flinders University, GPO Box 2100, Adelaide 5001, South Australia, Australia; E-Mail: julie.ratcliffe@flinders.edu.au

**Keywords:** smoking cessation, Greek-Australians, older people, socio-cultural influences, facilitator, barriers

## Abstract

Smokers of all ages can benefit by quitting, but many smokers continue to smoke. Older Greek-Australian smokers, one of the largest ethnic groups in Australia, have higher rates of smoking than other groups of older Australians. This qualitative study aimed to explore older Greek-Australians’ views about socio-cultural influences on their smoking. A snowball sampling technique was used to identify twenty Greek–Australian smokers (12 males and eight females), aged ≥ 50 years. They were recruited through the Greek Orthodox Community Center of South Australia (GOCSA). Qualitative data were collected using semi-structured face-to-face interviews. The audio-taped interviews were translated and transcribed, and then analysed using content analysis. Results suggested that smoking was considered as the “*norm*” by older Greek-Australian smokers. There were four groups embedded in the participants’ social networks that were reported to be important in relation to either encouraging smoking or, smoking abstinence. These support groups included: family members, friends, the Greek community, and physicians. Smokers’ family members (brothers) and friends were identified as facilitators of smoking whereas non-smoker family members (children and spouses) were reported as providing barriers to smoking. Different approaches were used by supporter groups to assist smokers to quit smoking—both planned and unplanned. Knowledge, planning of social and cultural supports, and addressing barriers to smoking cessation are a important part of health planning for older Greek-Australians. Social norms, including those arising from social interactions, and predisposing traits can influence smoking behaviour. Addressing the specific barriers to smoking cessation of older Greek-Australians is critical to addressing the risk for chronic disease in this group.

## 1. Introduction

Tobacco smoking remains the single greatest cause of preventable illness and death worldwide [1]. About 1.2 billion adults (one-third of the world’s adult population) used tobacco at the beginning of the present century and this number is predicted to increase to 1.6 billion by 2025 [1]. Current projections indicate that smoking will be responsible for more than 10 million deaths annually by 2030 [2,3,4]. The leading cause of death attributable to smoking is cardiovascular disease, followed by chronic obstructive pulmonary disease (COPD), and then lung cancer. Smoking in adults was responsible for about 22 percent of all worldwide deaths from cancer and for about 11 percent of all cardiovascular disease deaths [5]. Smoking in older people has been identified as a major risk factor in eight of the top 16 causes of death. For older smokers the risk of dying from heart attack is about 60 percent higher than older non-smokers [6].

Tobacco is a highly addictive drug, which makes quitting by older smokers especially difficult [7,8]. Misinformation and misconceptions among older people can lead them to believe that quitting is unnecessary or impossibly difficult. For example, many older smokers believe that smoking cessation in later life does not have any benefits for them, or they believe that some anti-smoking aids (such as nicotine-replacement patches) have risks attached [9]. Another common belief is that, because they have smoked for a long time, all the possible damage has already happened and so there would not be any benefit from quitting [8]. However, the benefits of quitting for older smokers have long been recognized [10] and the risk of death due to smoking is reduced within one to two years after quitting for older smokers compared to those who continue smoking [11]. Numerous studies have reported that older age is associated with a greater likelihood of cessation [12,13]. Conversely, it has been recognized that age is positively related to nicotine dependence [14] and this could represent a significant barrier to cessation for older smokers [12].

Numerous factors have been identified that may serve as either barriers or facilitators to smoking among older smokers. Cultural influences, including the social environment and attitudes of family, friends, and co-workers toward smoking within a cultural group may impact on smoking status [15]. Ongoing contact with smokers can help prevent older smokers from quitting. Some older smokers with serious established diseases have reported that friends and family have created barriers to smoking cessation [16,17]. For example, Honjo *et al*. reported that smoking by other family members can encourage a smoker to continue smoking [18]. The same findings have been found to apply in the work environment in regard to co-workers [19].

Smoking cessation for immigrant populations in general, and older immigrant populations specifically, is complicated. Older immigrants may experience additional barriers to behavior change not experienced by the general population.. For example, they usually have to access health services delivered by health providers who do not share their language or culture [20]. Some research suggests that they do not seek smoking cessation support from doctors because of feelings of mistrust or because of previous negative experiences with doctors [21]. Cultural values and social beliefs are important factors which affect the smoking status of older immigrants and which govern whether smokers continue this behaviour or succeed in quitting. A qualitative study among American-Indian smokers found that ritual smoking behaviour is affected by the socio-cultural context of participants such as the ceremonial use of tobacco among urban American Indian participants, and tobacco farming and cultivation [22]. In a similar study among African-American smokers, it was found that smoking was considered the norm and around eighty percent of participants overestimated the rate of smoking among the Black population [23]. Similarly, in another study among Vietnamese participants it was found that the cultural values of mental control and self-determination were the most important factors in their successful quitting of smoking [21].

Despite the decrease in the prevalence of smoking in the Australian population over the past two decades, smoking rates for some non-English-speaking background (NESB) groups remain high [24,25]. Greeks form an important ethnic group in Australia; Greek is the second most commonly spoken language at home in Australia [26], and is the fourth most frequently spoken language overall [27]. Both Greek migrants and their Australian-born children are eager to preserve their ethnic identity by and do this by speaking Greek at home, protecting Greek religious and social beliefs, and marrying within the same community [28].

Previous studies have shown a high smoking rate among Greek-Australians compared to other ethnic groups. In 1998, a household survey in the Marrickville Local Government Area revealed that smoking among Greek-Australian males was significantly higher than for the general population (43 percent compared to 23 percent). Other studies of smoking prevalence among Greek-born males have shown similar results [29]. The smoking rate among Greek-Australian older people is also higher than the average for other older Australians; it is approximately 18.4 percent for Greek-born Australians aged more than 70 years of age in comparison with Australian-born people in the same age group where the prevalence rate is 12 percent [30]. 

There are very few studies of smoking in the Greek-Australian community in general and there is no study on attitudes to smoking of older Greek-Australian people specifically. Considering the fact that Greeks form one of the main ethnic groups in Australia, and that smoking rates in older Greek-Australians are higher than the average rate for other older Australians, understanding socio-cultural influences on their smoking behavior could illuminate different facilitators of, and barriers to, smoking cessation for this population. Hence, this study sets out to explore the socio-cultural factors that can influence older Greek-Australians to smoke or abstain.

## 2. Methods

### 2.1. Design

A qualitative study was undertaken with twenty Greek–Australian smokers (12 males and eight females), aged ≥ 50 years in order to explore the socio-cultural factors that influenced their smoking consumption and smoking cessation behaviour. Participants were interviewed individually using a semi-structured, face-to-face, in-depth interview. 

### 2.2. Sample and Setting

This study involved adults aged 50 (51–79 years of age) or over who self-identified as Greek-Australians, who lived in metropolitan Adelaide, and were currently smoking (defined as having smoked at least 100 cigarettes during his/her lifetime and currently still smoking) [31,32]. The Greek Orthodox Community Centre of South Australia (GOCSA) acted as the centre for recruiment of participants. Smokers who met the inclusion criteria were invited to participate in the study. Those who either attended the centre as vistors or who worked as staff were included. We recruited older Greek-Australian smokers into the study using snowball sampling [33] where individuals identify potential participants known to them. To achieve data saturation, 20 smokers (12 males, eight females) were recruited [34]. Following approval from the GOCSA manager and informed consent from participants, face-to-face interviews were conducted at the centre in a suitably quiet room. The samples were collected in two sessions. Following the first ten interviews and subsequent review of transcripts in the first session it was determined that more data were required and a further ten smokers were recruited for a second session. 

### 2.3. Recruitment and Data Collection

After visiting GOCSA and obtaining information from the community managers, the researcher realized that most Greek-Australians, even though living in the Australian community, prefer to speak Greek. All written materials were offered in both Greek and English. These included the consent form, letter of introduction, and information sheet. To make the English written materials understandable and readable for the participants, they were translated into Greek by a bilingual translator. The translations were checked for accuracy by another translator. One of the translators also helped the researcher conduct the interviews. 

### 2.4. The Interview

The interviews were conducted by a researcher with experience in conducting semi-structured interviews. Face-to-face interviews that lasted 45 to 60 min were conducted. The interviews included open-ended questions aimed at exploring the participants’ experiences of socio-cultural influences on their smoking and smoking cessation attempts. Demographic information (age, marital status, education, employment status and, preferred language) was collected. Every interview started with an invitation to participants to speak freely about the role of friends, family and local community members played on their smoking behavior or quit attempts. An interview schedule was developed based on the relevant literature and the study’s research aims and questions. The interview schedule was developed through an iterative process (where interviews and analysis occured in parallel) [35].

Interviews were continued until data saturation was achieved ([36]; see Data Transcription and Analysis below). Interviews were conducted in the respondent’s preferred language (English or Greek). A nationally accredited translator was used where necessary. This person translated questions and prompts from English to Greek and responses from Greek to English. All interviews were recorded for later transcription. Thirty dollars reimbursement was offered to compensate the respondents for time and out-of-pocket expenses associated with their participation.

### 2.5. Data Transcription and Analysis

The interviews were transcribed by the researcher. Transcriptions were concurrent with data collection. To achieve transcription accuracy a copy of the text was provided and the transcriber listened to the audio recording a second or third time and corrected any errors. To understand how data saturation might apply to this study, the researcher reviewed the content of transcribed interviews and all the codes, concepts, categories, relationships and themes. The researcher concluded that there was an element of data redundancy and that the categories, themes and inter-relationships had been thoroughly described. At this point, the researcher felt that interviewing would not lead to new information or themes, and it was concluded that data saturation had been achieved.

Manual data analysis started immediately after transcription of each interview. The resulting text was analysed using content analysis to identify the final themes. Content analysis helps the researcher to ‘sanitize’ words into a number of content-related categories then count the number of instances that fall into each category [37]. It has been suggested that when classified into the same categories, words, phrases and the like come to share similar meanings [38]. The purpose of creating categories for content analysis is to provide a means of describing a phenomenon, to increase understanding and generate knowledge [39]. To create the final themes, two stages occur during the process of content analysis. At the first stage, less relevant passages and paraphrases with the same meaning will be skipped. To achieve greater similarity between the categorized meanings, similar paraphrases are bundled and summarized at the second stage [40].

All the texts were read and re-read closely by the researcher and the difference in coding was discussed with the supervisors and some of the codes were adjusted. The researcher continued recoding of the transcriptions and followed it to achieve a sufficient consistency with the coding system. This approach was used to code the rest of the interviews. After emerging the new codes, the coding system were adjusted again and based on the latest structure the transcribed interviews were read again. Through comparing and contrasting all the codes, the researcher decreased the number of original codes to find similarities. Codes with clear connections and relevance were clustered and allotted descriptive labels [39]. Codes were also analyzed in terms of their frequency so that their occurrence in each category could be specified and compared (e.g., “*five of seven interviewees have said…*”, “*the majority of the answers focused…*”). As is common in a content analysis, the written summary included details of the number of people who provided similar responses and qualitative quotes were used to exemplify each category of response [41]. The text extracted from the transcripts and categorized a similar answers to each question. A content analysis of the complete dataset was undertaken [41] and emerging themes were identified. The researcher used SPSS (version 20) to generate descriptive statistics to illustrate smokers’ education, age, and health status.

### 2.6 Ethical Considerations

This study was approved by the Social and Behavioural Research Ethic Committee (SBREC) of Flinders University, South Australia. The participants were provided with a consent form, an introduction letter, and an information sheet in both English and Greek versions. The aim of the study was clearly explained to the participants and they were aware that their participation in the study was voluntary. All participants were informed that their transcribed information would remain confidential. 

## 3. Results

### 3.1. Participants’ Characteristics

Twenty Greek smokers who were more than 50 years old were interviewed for this study (12 males and eight females). Their mean age was 64.6 years (SD = 9. 96 years). Most of the participants had completed high school (12) and most of them preferred to communicate in Greek (12). Twelve identified as pensioners. Most (13) were suffering currently from diseases such as cancer or heart disease (Table 1).

The majority of the participants (17) stated that they only smoked cigarettes. All of the participants started smoking when they were young. The mean age of smoking commencement was 19 years (SD = 3.72 years) and 14 of the respondents said that they started smoking when they still lived in Greece. The mean years of smoking was 45.5 years (SD = 10.8), and about half of the respondents (11) said that they had smoked for more than 50 years.

The mean number of cigarettes smoked each day was 16.5 (SD = 9.98). Fourteen of the participants indicated that they routinely smoked within 30 minutes of waking. Eleven of the participants mentioned that they had attempted quitting at least twice, with the maximum attempts 15. Among the 16 smokers who had tried to quit smoking, 11 of had quit for at least three months and the maximum was 20 years. Five of the participants had previously quit for less than two weeks. (Table 2).

**Table 1:** Personal characteristics of participants.

Participants Code/Gender	Age	Education Level	Preferred Language	Occupation	Health Status
1. Male	79	Primary school	Greek	Pensioner	Three heart attacks and bladder cancer
2. Male	71	Primary school	Greek	Fitter/welder/blacksmith (Pensioner)	No disease
3. Female	51	High school	English	Work in nursing home (GOCSA)	No disease
4. Male	76	Primary school	Greek	Farmer/ laying cement foundations (Pensioner)	Heart surgery twice (coronary obstruction)
5. Male	73	Primary school	Greek	Pensioner	Colon cancer
6. Male	73	Primary school	Greek	Pensioner	No disease
7. Male	74	Primary school	Greek	Gas company (Pensioner)	No disease
8. Male	74	Primary school	Greek	Assembler (pensioner)	Respiratory problems
9. Female	61	High school	Both English and Greek	^*^ Translator	Respiratory problems
10. Female	65	High school	Both English and Greek	Pensioner	High blood pressure and hyperthyroid
11. Male	66	High school	Greek and English	Taxi driver	Diabetes and sarcoidosis
12. Male	62	High school	Greek	Pensioner	Back pain
13. Male	51	High school	Greek	Taxi driver	No disease
14. Female	53	Bachelor degree	English	School teacher	High blood pressure
15. Female	70	High school	Greek	Pensioner	Osteoporosis

^*^ Participant was not a translator for the current study.

### 3.2. Who were the Supporters of or Barriers to Cessation

Four social groups have been identified in the data, based on the extracted themes, as either supporters of smoking cessation or presenting barriers to smoking cessation. These included: family members, friends, Greek community members and physicians. Although the degree of influence of these groups was not equal, it is useful to consider each separately because each exerted unique influence.

#### 3.2.1. Family Members

The most important influence factor on smoking was the perception that smoking cigarettes had become the “*norm*” within their social environment. The majority of participants (15) had at least one family member who was also a smoker. Fifteen participants had at least one child who was currently smoking or who had smoked in their lifetime. Most of their parents (16) had been smokers and over half of the respondents (11) lived in a family where one parent or both died due to smoking-related disease, such as cancer or heart disease.

All of the participants expressed a tendency to trust their family more than other social groups in relation to supporting their attempts to quit. When they were asked about the supportive role of family members, the majority of the respondents (16) mentioned that a close family member (wife or children) had advised them to quit smoking and/or to smoke outside. P4 (a 76-year-old male) explained how other smokers made judgements about his smoking status and the role his wife played in supporting him to quit:

**Table 2:** Smoking-related characteristics of participants. Fix text flow-symbols after text.

P.C	Kind of Smoking	Starting Age/Country	Years as a Smoker	Average of Cigarettes Daily	Approximate Starting Time after Waking up	Number of Quitting Attempts	Longest Episode of Quitting
1	Cigarettes	22/Greece	57	18–20	5 min	3	20 years
2	Rolling Tobacco	17/Greece	54	20–25	30 min	0	--
3	Cigarettes	27/Australia	24	12	30 min	4	2 weeks
4	Cigarettes	16/Greece	58	25	2–3 h	1	2 years
5	Rolling Tobacco	12/Greece	61	2–3	30 min	1	3 days
6	Cigarettes	22/Greece	51	10–12	20 min	1	^**^ 2 weeks
7	^*^ Cigarettes	19/Greece	55	25–30	20–30 min	0	--
8	Cigarettes	24/Greece	50	20	30 min	1	^***^ 4 days
9	Cigarette	16/Greece	45	6–10	10 min	10	1 year
10	Cigarette	20/Greece	47	4	10 min	10-15	6 months
11	Cigarette	15/Greece	49	30	5 min	4-5	9 months
12	Cigarette	14/Greece	48	18–20	20 min	2-3	6 days
13	Cigarettes	19/Australia	32	25	1–2 h	0	--
14	Cigarettes	18/Australia	30	10	1 h	3-4	11 years
15	Cigarettes	25/Greece	45	2–3	No	2	^****^ 3 years
16	Cigarettes	17/Greece	55	25	15 min	2	2 years
17	Rolling Tobacco	18/Australia	35	30	5 min	3	5 years
18	Cigarette	19/Greece	50	10	30 min	4	^****^ 9 months
19	Cigarette	20/Australia	30	2–3	No	1	3 months
20	Cigarette	20/Australia	35	3–4	No	0	--

^*^ He started with rolling tobacco but now smokes cigarettes; ^**^ Due to getting cold; ^***^ Due to stay in hospital; ^****^ Due to pregnancy.

*“My wife kept telling me to stop smoking but I know a lot of people that used to smoke three packets a day and don’t know how to quit and they say to me well, you don’t smoke a lot just three or four cigarettes a day — why do you want to stop? You know they smoke a lot more than I do and smoking hasn’t really affected me, that is why”.*

Some of the interviewees (five) said that they had reached an agreement with their family to smoke only outdoors. However, their families were still not happy with their smoking. Two of the respondents reported that their children had drawn up a plan to help them to quit smoking. One stated his children advised him to reduce his smoking by only smoking three cigarettes a day, one in the morning-after coffee, one in the afternoon after lunch, and finally one cigarette after coffee, before going to the bed. P1 (a 79-year-old male) described the plan his son had drawn up to help him to quit:

*A lot of friends are asking me why I smoke. My son is 55 years old now he used to smoke and gave up and he keeps telling me to do the same. One time he said to me. I will bring you three cigarettes every morning before I go to work:— one for the morning, one for lunch and one for after tea.*

Most of the interviewees stated they felt uncomfortable when they were under pressure to quit from family members and one participant reported that her sister ridiculed her and made her angry, and so she stopped listening to her advice about quitting smoking. She said she wanted advice to be given respectfully. P3 (a 51-year-old female) highlighted the negative influence of constant “*nagging*”:

*Oh. Yes. She will support me. She will support me. Absolutely, but her support is nagging and I don’t want to be nagged. If you are going to nag me I will switch off. I’ll walk away I’ll go and light a cigarette. I’ll do the upset to why to telling me too if you are nagging me. You know it is like a child—the mother tells him not do something so the child then does it.*

Stressful family circumstances also provided barriers to smoking cessation attempts. Of the 16 participants who had attempted to quit, three of them reported that their wife or child’s death was the main reason for their smoking relapse. Six of respondents stated that visiting other family members who smoke, or having family-related stress were common causes of smoking relapse. 

P11 (a 66-year-old male) had attempted to quit five times. His wife’s death made him smoke again to ameriolate perceived levels of stress he was experiencing. Here P11 highlighted the role of adverse life events and stress as a barrier to smoking cessation:

*Because your life is full of ups and downs happening every day. You have to cope with a lot of problems every day. That is your lifestyle and then you go somewhere, sit down with smokers and have a coffee. You have stress because of your family or your kids and they all push you to do something…*

#### 3.2.2. Friends

Six of the respondents stressed that they did not wish to receive any support to quit from their friends and if their friend did try to help them, they would reject their offer of support. Their reasoning was related to “*individual freedom*” as reflected in comments like “*I don’t like to be dictated to by anybody*”. For them smoking was a personal issue. P2 (a 71-year-old male) stressed his personal freedom to smoke and said that advice from friends about giving up would be unwelcome:

*I don’t want to blame you if you want to help me or if anyone else wants to help me. But it won’t work with me. I am happy that I can express my freedom to smoke in a free country.*

Eight of the respondents declared that their friends advised them to quit smoking, but did not offer serious support. However, some responses indicated that friends do have a potential to support cessation. For example, 12 of the interviewees claimed that when they were with non-smoking friends, they were reluctant to smoke. P20 (a 50-year-old female) highlighted the positive role of non-smoker friends:

*Yes less. When you are with a person who doesn’t smoke you smoke less because she doesn’t smoke.*

Conversely, in some cases friends were described as a barrier to smoking cessation attempts. Of the 16 participants who had attempted to quit, six of them reported that visiting smoking friends, was the main reason for relapse or encouraged them to smoke more. 

P9 (a 61-year-old female) was an interpreter with a respiratory problem. She had been a smoker since the age of 16 but she had stopped many times. P9 described her many quit attempts and barriers to her smoking cessation:

*If you are with someone that smokes it is easy to start again by having one and then another and from then on go and buy a packet.*

P12 (a 62-year-old male) was a pensioner and highlighted the role of social networks, especially the pressure of friends, as a barrier to quitting smoking:

*It was because of the company. I have two or three friends. They offer me cigarettes and I say I am quitting but they urge me to take a cigarette. They tell me to take just this one and then I start again. It is not because of weak willpower*

#### 3.2.3. Greek Orthodox Community Centre

The majority of the participants (16) were members of GOCSA, and they engaged other members of the community at least once a week. When they visited other members of their community, they preferred to associate with their closest friends. Whether they were smokers or not was not a criterion for friendship. P6 (a 73-year-old male) mentioned the importance to him of friendship with other community members:

*I like to sit with my friends who are very close to me. It doesn’t matter who is a smoker or who is not.*

The Greek community has been reported as a place to share their experiences and culture. As smoking was a “*norm*” in their culture, being a smoker or non smoker was not an important issue in their visits in the community. In another word, membership of this group was neither a barrier or facilitator to smoking cessation. Advice given to others on their smoking behavior, at the community level, was rare because the participants reported that they didn’t like to interfere in other people’s affairs.

#### 3.2.4. Physician 

In some cases participants reported that their doctor was their main source of knowledge about the relationship between smoking and ill-health. 

P11 (66-year-old male) stated he was reluctant to take his doctor’s advice to cease smoking and exercise. He was uncertain whether the doctor was linking smoking to the health problems he was experiencing and he was reluctant to inform his doctor about the extent of his smoking:

*Yes, my doctor told me that it could be because of that [smoking]. He told me that exercise is good but I have got some inflammation and I need to be careful* […] *The doctor tells me “don’t smoke”; he doesn’t know how many cigarettes I smoke but he tells me that it is not good.*

The majority of participants who were willing to receive support in their attempts to stop smoking would prefer it to be administered by their doctor. P17 (53-year-old male) expressed a high level of intention to quit and had discussed the new technological aids with his doctor:

*Yes I would accept and I am talking with my doctor at the moment. I asked him about electronic tobacco and I am thinking a lot about giving up. I want to start to cut down and you know I have heard about electronic cigarettes and a lot of people have used it and it makes you more healthy.*

However, all of the participants mentioned that they had not received any useful support to quit smoking. Whenever they received advice from their doctor in relation to smoking cessation, they felt that the advice was not practical. P9 (a 61-year-old female) had a family where most members smoked cigarettes. Her brother was a heavy smoker and her two sisters, in Greece and Sydney, also smoked. P9’s doctor told her to stop smoking but did not offer any supportive program:

*My doctor just tells me off and says “you should just quit” but I haven’t listened to him — I stop and then start again usually.*

Four of the participants stated that their doctor had prescribed nicotine patches for them, but they could not continue this treatment due to side-effects such as abdominal pain, finger swelling and other problems. P5 (a 73-year-old male) describes his problems with side-effects:

*I tried once [to give up] and went to the doctor and he gave me some tablets to help with stop smoking. After I took them my hands swelled up and I couldn’t get my ring off my finger; I had to cut the ring off. I went to the Queen Elizabeth hospital and I saw the doctor and he gave me three small tablets.*

A few of the respondents reported that that is too late to seek help from the doctor. P14 (a 53-year-old female), had attempted to quit smoking three times and the longest quit episode lasted 11 years. She separated from her husband and she was then surrounded by people who smoked. She believed that educational programs were appropriate for younger people, not for older adults:

*The only thing that I would say is that young people today are more willing to get educated, you know when they are 20 or 25, but older people don’t care about that so much. Even they go to the doctor and the doctor tells them “you are in danger and you have to stop”, most of them don’t care. That is my belief. I know probably I smoke and I’m guilty too but it’s just the way that we think, like tomorrow is another day.*

Advice from their doctor lacked credibility in the case where their doctor smoked:

*“When I went to my doctor’s office, he told me not to smoke but then I saw he was smoking”*.

(P5)

## 4. Discussion

The role of social networks, including family members, friends and others is very important to smoking cessation and continuation [42]. The results of our study concur with the findings of another study conducted among Asian-Americans [43]. Here there was a positive relation between smoking more cigarettes and having friends that smoked. This study also showed if a person had a father who smoked they were at greater risk of smoking. Similarly, a previous study among Greek teenagers showed that smoking consumption was strongly influenced by family and friends. The results showed that around 30 percent of respondent’s cigarette consumption was related to family smoking behavior [44]. In another study among UK Bangladeshi and Pakistani communities, results showed that the main perceived barrier to quitting included being tempted by others [45]. Another study found that having fewer friends and family members who smoked was an associated factor in successful cessation, at least temporarily (for six months) among older smokers [46]. 

The effect of other family members and friends on smoking status is not always considered as a barrier and sometimes can be supportive to those quitting. In a study among smokers in the US, the results showed that social support (from doctors, friends, and family) was the main factor both motivation to quit [47]. 

Our study confirmed that smoking is a socially and culturally accepted activity among older Greek-Australians and every smoker interviewed had at least one other smoker in his/her family. Smoking was accepted as the norm in this particular group. There was no blame associated with smoking and indeed there seemed to be a positive attitude towards smoking among the Greek community. Consistent with this, a previous study in Greece also demonstrated that anti-smoking policies had a low chance of success because smoking was socially and culturally accepted among Greek people. Other factors like a low tobacco price and farming of tobacco in some regions of Greece assisted in creating a pro-tobacco norm among Greek people [48]. The pro-smoking culture in Greece may influence behaviours laid down in Greek youth by enhancing pro-smoking beliefs [49].

According to the theory of planned behaviour perceptions of what is normal (*i.e.*, the subjective norm) can contribute to both cessation and continuation of smoking. Individuals with a more positive attitude towards smoking cessation generally report a subjective norm that supports cessation [50]. This subjective norm describes cessation as valued by important others including family and friends. The extent of influence of this norm is, however, constrained by an individual’s motivation to comply with the opinion of others [51]. In the current study, complying was not universally endorsed by smokers.

In a study among Greek smoker and non-smoker adolescents, to understand views about second hand smoke (SHS), the results showed that smoking was perceived to be both an inherent part of socializing and highly addictive. The participants considered smoking cigarettes in public places as a “*right*”. The participants believed that smokefree legislation would not be successful in Greece because Greeks are inherently non-conformist, rebellious, free spirited, libertarian, individualistic and even somewhat selfish and egotistical. The results also showed that smoking was generally perceived to be a widespread and represents normal behaviour among Greek adults and young people, and many non-smokers would inevitably become smokers over time. This result, together with data reported here highlights the dominant belief that smoking is normal, particularly when socializing with friends [52]. 

A lack of anti-smoking advice by physicians or other health professionals has been highlighted in our findings with older Greek-Australian smokers. Factors such as old age and addiction to nicotine were reported as important and affects readiness to receive anti-smoking service delivery and advice. Several participants also had a negative attitude to doctor’s advice to quit smoking; for doctors who smoked the participant saw incongruence between behaviour and recommendations. 

Some previous studies reported that older smokers are unconvinced about the effectiveness of anti-smoking advice from a doctor [53,54]. According to the International Guidelines, physicians should assess the smoking status of their patients and provide brief supportive advice to smokers about quitting [55]. In one study it was found that few UK Bangladeshi and Pakistani participants had sought advice from health services, or received cessation aids such as nicotine replacement therapy (NRT); moreover, family doctors were not viewed as accessible sources of advice on quitting [45]. 

Some opportunities for intervention have been suggested aimed at enhancing motivation to quit and supporting cessation efforts. Such interventions may include educational programs on health impacts of smoking and cessation, increased training and support of healthcare providers to give brief advice, formal and self-help treatment opportunities, and adjunct pharmacotherapy for nicotine withdrawal [53]. Taking this further, special strategies should also be considered for older smokers. Health professionals should particularly emphasize the health consequences of smoking becausewe found that those who believed that smoking was affecting their current health status, and would likely cause them serious health problems in the future, were at a higher stage of readiness to quit. In light of this, health professionals should treat their older patients in the same way as their younger-adult patients. Guidelines recommend that practitioners ask *all* patients about their smoking habits; advise *all* smokers about the risks; assess their willingness to change; assist them in setting a quit timeline; provide resources such as NRT, and arrange a follow-up appointment to monitor progress. In addition to using this standard approach, clinicians may be able to identify and incorporate strategies for addressing social normative barriers to quitting [56].

## 5. Conclusions

The present study has identified many key points in understanding the unique socio-cultural characteristics of older Greek-Australian smokers that influence smoking prevalence and and smoking cessation. We found that for this group smoking has been accepted as a social and cultural norm. The most frequent responses concerning guidelines or potential facilitators of cessation suggested that Greek community members, friends, family members and doctors are all potential facilitators that could support smokers to quit smoking. The role of family members stands out as the main factor that could be effective with this group. The role played by family members in causing them to relapse after smoking cessation was highlighted. Older Greek-Australian smokers expressed a tendency to trust their family more than doctors or friends as supporters in attempts to quit. 

The Greek community is recognised as a potential place to engage older Greek-Australians and is characterised by people making regular and continuing visits. Physicians were reported as the main and valid source of smoking-related health knowledge although they were uniformly trusted or listened to. Some smokers followed their physician’s recommendations, however, there was report of very little practical advice provided by physicians to support smokers to quit. In some cases, participants justified their smoking behaviour in relation to their cultural beliefs about smoking. Future cessation research should focus on efforts to better understand the characteristics of minority group smokers and continue work that helps us to unravel the complicated nature of smoking cessation.
